# Influence of subinhibitory antifungal concentrations on extracellular hydrolases and biofilm production by *Candida albicans* recovered from Egyptian patients

**DOI:** 10.1186/s12879-019-3685-0

**Published:** 2019-01-16

**Authors:** Houdaii H. El-Houssaini, Omnia M. Elnabawy, Hebatallah A. Nasser, Walid F. Elkhatib

**Affiliations:** 1 Department of Microbiology and Public Health, Faculty of Pharmacy, Heliopolis University for Sustainable Development, 3 Cairo-Belbeis Road, El Horreya, Cairo, 11788 Egypt; 2 Department of Clinical Pathology, Faculty of Medicine, Ain Shams University, Abbassia square, Cairo, Egypt; 3 Department of Microbiology and Immunology, Faculty of Pharmacy, Ain Shams University, African Union Organization St., Abbassia, Cairo, 11566 Egypt; 4 Department of Microbiology and Immunology, School of Pharmacy and Pharmaceutical Industries, Badr University in Cairo (BUC), Entertainment Area, Badr City, Cairo, Egypt

**Keywords:** Virulence, Antifungals, Subinhibitory concentrations, Hydrolases, Biofilm

## Abstract

**Background:**

Extracellular hydrolases (phospholipase, aspartyl protease and haemolysin) and biofilm production are considered as major virulence factors of the opportunistic pathogenic fungus *Candida albicans*. However, the impact of antifungal therapy on such virulence attributes is not well investigated. The common antifungal agents may disturb the production of secreted hydrolases as well as biofilm formation. Accordingly, this study addressed the effect of subinhibitory concentrations (sub-MICs) of selected antifungal agents on some virulence factors of *C. albicans* clinical isolates.

**Methods:**

*C. albicans* isolates (*n* = 32) were recovered from different clinical samples and their identification was confirmed to the species level. Antifungal susceptibility profiles of isolates were determined against (nystatin, fluconazole and micafungin) and minimum inhibitory concentrations (MICs) were interpreted according to Clinical and Laboratory Standards Institute guidelines. Virulence determinants comprising secreted hydrolases (phospholipase, aspartyl protease and haemolysin) and biofilm formation were investigated in the presence of the sub-MICs of the tested antifungal agents.

**Results:**

Treatment of clinical *C. albicans* isolates with subinhibitory nystatin, fluconazole and micafungin concentrations significantly decreased production of extracellular hydrolases. Nystatin had the greatest inhibitory effect on phospholipase and aspartyl protease production. However, micafungin showed the highest reducing effect on the hemolytic activity of the treated clinical isolates. Moreover, nystatin and micafungin, but not fluconazole, had a noticeable significant impact on inhibiting biofilm formation of *C. albicans* clinical isolates.

**Conclusion:**

Our findings highlighted the significant influences of commonly prescribed antifungal agents on some virulence factors of *C. albicans*. Accordingly, antifungal therapy may modulate key virulence attributes of *C. albicans*.

**Electronic supplementary material:**

The online version of this article (10.1186/s12879-019-3685-0) contains supplementary material, which is available to authorized users.

## Background

*Candida albicans* is a member of the human normal microbiota, colonizing the oral cavity and the gastrointestinal and genitourinary tracts in most of healthy individuals [1]. When host defenses become compromised due to hospitalization, treatment with antibiotics, surgery and the use of catheters as well as prosthetic devices [2], *C. albicans* can cause symptomatic infections, ranging from superficial skin or mucosal infections to life-threatening systemic ones [3]. The switch from commensalism to pathogenesis depends on both fungal and host factors. Some pivotal fungal virulence factors involve dimorphism, adhesion, secretion of hydrolytic enzymes and biofilm formation [4], while host factors include immunodeficiency, epithelial damage or microbial dysbiosis [5]. Phospholipases are responsible for lipids digestion for nutrient acquisition, adhesion to host tissues, synergistic interactions with other enzymes, nonspecific hydrolysis, initiation of inflammatory processes by provoking cells of the immune system and self-defense [6]. Aspartyl proteases are in control of several steps in innate immune evasion and they degrade proteins related to immunological defense such as antibodies, complement and cytokines, allowing the opportunistic fungus to escape from the first line of host defenses [7]. Haemolysin is crucial to derive iron from the host’s erythrocytes, which is considered one of the essential micronutrients for the opportunistic pathogen to thrive and survive in their hosts [8, 9]. Biofilms of *C. albicans* are multicellular communities of yeast, pseudohyphae and hyphae coated by extracellular matrix, which form upon adherence to biotic or abiotic surfaces. Living within a biofilm has many advantages, which include protection against the environment, resistance to physical and chemical stresses, metabolic cooperation and joint regulation of gene expression for the associated microorganisms [10].

Several studies have attempted to explain the mechanisms of *C. albicans* pathogenicity. Nevertheless, the effect of different antifungal agents on the production of fungal virulence factors may still need further investigation [11]. Some investigators have reported that exposure to certain antifungal agents may lead to reduced virulence attributes by some tested yeasts [11, 12]. Represented by nystatin, polyenes demonstrate fungicidal activity mostly through targeting ergosterol in the fungal cell membrane resulting in a complex, which is able to disrupt membrane integrity and eventually leading to leakage of vital cellular contents [13]. Azoles, represented by fluconazole, which is fungistatic [14], exert their action through blocking the cytochrome P450 enzyme 14-α demethylase, which catalyzes the conversion of lanosterol to ergosterol, thereby arresting ergosterol biosynthesis and affecting the fungal membrane integrity [15, 16]. The echinocandins, represented by micafungin, target the enzyme glucan synthase, which is responsible for the synthesis of β-1,3 glucans [17], resulting in a weakened fungal cell wall and leading to cell lysis and death [15, 18].

Despite the fact that candidiasis can be treated with polyenes, azoles and echinocandins, once administered, the concentration of these antifungal agents tend to be reduced due to drug metabolism/tissue bioavailability or the presence of biofilms. The current study aimed to investigate the impact of different antifungal agents representing three major classes (polyenes, azoles and echinocandins) on secreted hydrolases and biofilm formation by clinical isolates of *C. albicans*.

## Methods

### *Candida albicans* isolates

In the present study, *C. albicans* isolates (*n* = 32) were collected from different clinical specimens: (62.5% vaginal swabs, 12.5% urine, 12.5% sputum and 12.5% miscellaneous sources) at the main Microbiology laboratory in El-Demerdash hospital, Faculty of Medicine, Ain Shams University (Cairo, Egypt). Isolates were recovered on Sabouraud’s dextrose agar (SDA) (Oxoid Ltd., United Kingdom) and incubated aerobically for 24 h at 37 °C. Before being investigated, all isolates were subcultured twice on SDA from the stock cultures stored at − 80 °C to guarantee viability and purity. *C. albicans* isolates were identified to the species level as described previously by [19, 20].

### Antifungal susceptibility testing

In vitro antifungal susceptibility testing was conducted as proposed by the Clinical and Laboratory Standards Institute (CLSI) M27-A3 protocol [21] against nystatin (NYS), fluconazole (FLU) and micafungin (MCF) (Sigma- Aldrich Chemical Corporation, St. Louis, MO, USA). In 96-well round bottom microtiter plates (CITOTEST, Haimen, China), two-fold serial dilutions of these antifungal agents were performed with RPMI-1640 medium (Hi-Media, Mumbai, India), buffered with 0.165 M 3-N-morpholinepropanesulfonic acid (MOPS), pH 7.0. Inoculum preparation was obtained from a 24 h SDA culture incubated at 35 °C; the yeast cells were suspended in RPMI-1640 medium and turbidity was adjusted to about 1–5 × 10^3^ cells/mL. *C. albicans* ATCC 90028 was used as a quality control strain for the experiment. The susceptibility profiles of the tested isolates were interpreted as recommended by the CLSI protocol where the MIC values were determined visually after 24 h incubation by comparing the growth in the wells with the antifungal agent with the drug-free control. Turbidity was scored from 0 to 4 with 0 for visually clear, 1 for slightly hazy growth, 2 for a noticeable reduction in turbidity (around 50%), 3 for a slight reduction in turbidity and 4 for no reduction in turbidity in comparison to that of the drug-free control well. For amphotericin B, the endpoint is defined as the highest dilution that inhibits visual growth or an endpoint score 0 and score 2 endpoint for azoles and echinocandins [21]. MICs were validated after a second experiment performed under the same conditions with the same MICs verified for each isolate.

### Preparation of yeast suspension & exposure to antifungal agents prior to extracellular hydrolases assay

Inoculum preparation was obtained from an 18–24 h SDA subculture incubated at 35 °C, after which cells were suspended in sterile phosphate-buffered saline (PBS, pH 7.4) to reach an optical density equivalent to 0.5 McFarland standard at 520 nm. Aliquots (4 mL) from this fungal suspension were added to a tube containing 4 mL of RPMI-1640 medium (control) and 4 mL of RPMI/antifungal solution (test) in which the antifungal concentrations were equal to the MIC value of the tested antifungal agent, giving a cell suspension of 10^6^–10^7^ cells/mL in each assay tube. The tubes were then incubated for a period of 1 h for phospholipase [22] and haemolysin [11] assays and another set of tubes incubated for 18 h for aspartyl protease assay [23] at 37 °C in a shaking incubator (A&B Lab Co. LTD., TZH United Kingdom). The antifungals were washed by two cycles of dilution using sterile PBS and centrifugation for 10 min at 3000×g after the exposure time, followed by complete decantation of the supernatant. The pellets were resuspended in 5 and 2.5 mL of sterile PBS, respectively, which ensures 10,000-fold antifungal concentration reduction [24, 25]. Therefore, this method virtually excludes any ‘carry-over effect’ of the antifungal agent following its removal. Viable counts of the control and test were conducted by the spreading plate technique after drug removal and control suspensions were reconstituted as needed to obtain a cell density equal to the test. An aliquot of the resulting suspension was gently vortexed for 1 min, serially diluted and inoculated using a sterile spreader onto SDA plates. After incubation for 48 h at 37 °C, the *Candida* colony-forming units/mL (CFU/ml) were counted. Remaining fungal suspensions were resuspended as required to obtain a standard cell concentration (1× 10^8^ cells/mL).

### Phospholipase assay

*C. albicans* isolates’ phospholipase activities were determined by a previously described method [26, 27]. Aliquots (5 μL) of standardized fungal inoculum of the previously mentioned antifungal-treated and control suspensions were spot inoculated aseptically onto egg yolk agar plates, which were left to dry out and then incubated at 37 °C for 48 h. The plates were checked for the appearance of precipitation zone around the colonies, which denotes phospholipase activity. The phospholipase index (P_z_) was calculated using the ratio between the diameters of the precipitation zone and the colony indicating the phospholipase production intensity [26, 28]. Each assay was carried out in quadruplicate on two individual experiments. *C. albicans* ATCC 10231 was used as positive control. Results for this test were expressed as the percentage reduction in phospholipase production applying the following formula:

$$ \mathrm{Phospholipase}\ \mathrm{reduction}\%=1\hbox{-} \left[\left({\mathrm{P}}_{\mathrm{z}}\mathrm{assay}/{\mathrm{P}}_{\mathrm{z}}\mathrm{control}\right)\right]\times 100 $$

### Aspartyl protease assay

*C. albicans* isolates’ aspartyl protease activities were evaluated by a previously described method [27, 29]. Aliquots (5 μL) of standardized fungal inoculum of the previously mentioned antifungal-treated and control suspensions were spot inoculated aseptically onto 1% *w*/*v* bovine serum albumin (Levochem, N.Y., U.S.A.) agar plates, which were left to dry out and then incubated at 37 °C for 5 days. Further proteolytic activity was stopped using 20% w/v trichloroacetic acid (S D Fine-Chem Limited, Mumbai, India) and plates were stained with 1.25% w/v amidoblack (Hi- Media, Mumbai, India) and checked for the presence of proteolysis zone around the colonies, which was not stained with amidoblack. The protease index (Pr_z_) was measured in terms of the ratio between the diameters of unstained zone and the colony [23]. Each assay was conducted in quadruplicate on two individual experiments. *C. albicans* ATCC 10231 was the positive control strain for the experiment. Results for this test were expressed as the percentage reduction in aspartyl protease production applying the following formula:

$$ \mathrm{Aspartyl}\ \mathrm{protease}\ \mathrm{reduction}\%=1\hbox{-} \left[\left(\mathrm{Prz}\ \mathrm{assay}/\mathrm{Prz}\ \mathrm{control}\right)\right]\times 100 $$

### Haemolysin assay

*C. albicans* isolates’ haemolytic activities were investigated on blood SDA plates by the method described by [27, 30]. Aliquots (5 μL) of standardized fungal inoculum of the previously mentioned antifungal-treated and control suspensions were spot inoculated aseptically onto the blood SDA plates, which were left to dry out and then incubated at 37 °C for 48 h under 5% CO_2_. The plates were checked for the existence of haemolysis zone around the colonies, which reveals haemolytic activity. The haemolytic index (H_z_) was calculated in terms of the ratio between the diameters of translucent halo and colony [23]. Each assay was carried out in quadruplicate on two individual experiments. *C. albicans* ATCC 90028 served as a control strain. Results for this test were expressed as the percentage reduction in haemolysin production applying the following formula:

$$ \mathrm{Haemolysin}\ \mathrm{reduction}\%=1\hbox{-} \left[\left({\mathrm{H}}_{\mathrm{z}}\mathrm{assay}/{\mathrm{H}}_{\mathrm{z}}\mathrm{control}\right)\right]\times 100 $$

### Biofilm production assay

Biofilm production was screened by the microtiter plate method as modified after [27, 31, 32]. In flat bottom microtiter plates (Hyundai Micro Co., LTD., Korea), 180 μL of Sabouraud’s dextrose broth (SDB) (Oxoid LTD., U.K) supplemented with 8% *w*/*v* glucose (ADWIC, Qalyubiah, Cairo, Egypt) were inoculated with 20 μL of a standardized fungal inoculum prepared in sterile saline (pH 7.4), which had its turbidity adjusted to approximately 3 × 10^7^ CFU/mL. Plates were then incubated at 37 °C for 24 h without agitation, washed with distilled water and then stained for 45 min with 0.4% w/v aqueous crystal violet solution. Plates were washed three times with sterile distilled water and immediately destained with 200 μl of 95% ethanol for 45 min. Afterwards, 200 μL of the destaining solutions were transferred to a new plate and the OD was measured with ELISA reader (Euroclone BIOTEK, Italy) at 590 nm [31, 32]. Biofilms were quantified in terms of the absorbance (color intensity) of the crystal violet solution trapped by the biofilms formed in the wells. *C. albicans* ATCC 10231 was the positive control strain for biofilm production and the assay was conducted in triplicates for each isolate. The OD cut-off value (ODc) for biofilm formation was calculated using the following formula: Average OD of the yeast-free negative control + 3× standard deviation (SD) of negative control [27]. The isolates were then classified as strong, moderate, weak and non-biofilm producers [33].

### Effect of antifungals’ sub-MICs on biofilm production

*Candida albicans* isolates which exhibited biofilm producing ability were grown in a tissue culture plate (SPL Life Sciences, Co., Ltd., Korea) in the presence of 0.5 × MICs of test antifungal agents as described in the modified method of Ramage et al. [34]. Volumes of 1 mL of test agents (2 × MIC) in RPMI 1640 medium were added to each well of the tissue culture plate. Wells with only medium were used as control (untreated) wells. Subsequently, 1 mL of standardized yeast cell suspension was added and the plate was incubated for the development of biofilms at 37 °C for 48 h. Results for this test were presented as the percentage of biofilm formation inhibition applying the following formula:

$$ \mathrm{Biofilm}\ \mathrm{reduction}\%=\left[1\hbox{-} \left({\mathrm{OD}}_{\mathrm{assay}}/{\mathrm{OD}}_{\mathrm{control}}\right)\right]\times 100 $$

### Scanning electron microscopy for biofilm production

Scanning electron microscopy was undergone for *C. albicans* biofilms in absence and presence of sub-MICs of the test agents. Biofilms were grown using the aforementioned method of Ramage et al. [34]. Preparation of test biofilms for SEM examination was attained as described previously by [27, 35]. Biofilm assay medium was aspirated and wells were washed twice with PBS (pH 7.4) so that the non-adherent *Candida* cells were removed. Biofilms were then fixed by adding glutaraldehyde solution (2.5% *v*/v in 0.1 M phosphate buffer, pH 7.4) and stored overnight at 4 °C, which was decanted afterwards and biofilms were washed with distilled water and then dehydrated using a graded series of ethanol (25, 50, 75 and 100%) and air dried for 20 min. The bottoms of the wells were cut for consequent SEM imaging.

### Statistical analysis

The SPSS version 22.0 statistical software (SPSS Inc., Chicago, Il, USA) was used to compute the present study’s statistical analyses. The data were found not to be normally distributed by applying the Shapiro-Wilk test for normality assumption and hence non-parametric tests were conducted. Statistical differences in the data obtained from the treated and untreated groups were analyzed using Friedman’s test; two way analysis of variance by ranks. Moreover, pairwise comparisons (using Friedman’s test) were computed, which treat one group as a control (unexposed to antifungal agent) and compare the other groups (exposed to antifungal sub-MICs) against it. A *p*-value of less than 0.05 was considered statistically significant.

## Results

Majority of the *C. albicans* isolates was obtained from vaginal swabs (62.5%), followed by urine (12.5%), sputum (12.5%) and miscellaneous samples (drain 6.25%, pus 3.12%, and blood 3.12%). Upon inoculation in human serum, *C. albicans* isolates produced germ tubes when examined under light microscope after being incubated at 37 °C for 2–4 h. When subcultured on *Candida* Chromogenic agar (Conda Laboratorios, Madrid, Spain), *C. albicans* isolates showed smooth green colonies after incubation at 37 °C for 48 h.

Concerning the antifungal susceptibility testing**,** all clinical isolates of *C. albicans* were sensitive to NYS (MIC ≤4 μg/ mL) and MCF (MIC ≤0.25 μg/mL). However, 100% showed resistance to FLU (MIC ≥64 μg/mL). An additional table shows these results in more details [Additional file 1].

Effects of the test antifungal agents (NYS, FLU and MCF), which imply different mechanisms of action, on extracellular hydrolytic (phospholipase, aspartyl protease and haemolysin) activities are summarized in Table 1. Concerning phospholipase, there was a significant difference (*p* < 0.05) in the P_z_ values represented by the control (untreated) and test *C. albicans* isolates (treated). The median extracellular phospholipase activity (P_z_) for the *C. albicans* isolates unexposed to antifungal treatment (control) was 1.0650 (range 1.0000–1.2634). There was a significant (*p*< 0.05) inhibition in the extracellular phospholipase activities after exposure to nystatin and micafungin with median P_z_ values of 1.0176 (4.45% reduction) and 1.0251 (3.75% reduction), respectively. Regarding aspartyl protease, there was a significant difference (*p* < 0.05) in the Pr_z_ values represented by the control (untreated) and test *C. albicans* isolates (treated). The median extracellular aspartyl protease activity (Pr_z_) for the control *C. albicans* isolates unexposed to antifungal treatment was 1.2628 (range1.0000–1.8000). There was a significant (*p*< 0.05) inhibition in the extracellular aspartyl protease production after exposure to nystatin and micafungin with median Pr_z_ values of 1.0556 (16.41% reduction) and 1.0580 (16.22% reduction), respectively. Concerning haemolysin, there was a significant difference (*p* < 0.05) in the H_z_ values represented by the control (untreated) and test *C. albicans* isolates (treated). The median extracellular haemolysin activity (H_z)_ of the control *C. albicans* isolates was 1.6814 (range 1.4296–2.0000). There was a significant (*p* < 0.05) inhibition in the extracellular haemolysin activities after exposure to nystatin and micafungin with median H_z_ values of 1.4736 (12.36% reduction) and 1.4272 (15.12%), respectively. Interestingly, exposure to fluconazole revealed a significant (*p* < 0.05) suppressive effect on the production of phospholipase, aspartyl protease and haemolysin with median values of 1.0282 (3.46% reduction), 1.0654 (15.63%), 1.4519 (13.65% reduction), respectively, despite the fact that *C. albicans* isolates showed resistance to fluconazole.

**Table 1:** Variations in extracellular hydrolytic activities in *Candida albicans* isolates exposed to antifungal sub-MICs

Virulence factor		Control	NYS	FLU	MCF
Phospholipase (P_Z_)	Median	1.0650	1.0176	1.0282	1.0251
	Range	1.0000–1.2634	1.0000–1.2152	1.0000–1.1944	1.0000–1.2347
	% Reduction	–	− 4.45%	− 3.46%	− 3.75%
	*p*-value		**0.010**	**0.047**	**0.022**
Aspartyl protease (Pr_z_)	Median	1.2628	1.0556	1.0654	1.0580
	Range	1.0000–1.8000	1.000–1.2000	1.0000–1.2667	1.0000–1.3333
	% Reduction	–	−16.41%	−15.63%	−16.22%
	*p*-value		**0.001**	**0.007**	**0.003**
Haemolysin (H_Z_)	Median	1.6814	1.4736	1.4519	1.4272
	Range	1.4296–2.0000	1.1667–1.8750	1.1574–1.8571	1.1309–1.8571
	% Reduction	–	−12.36%	−13.65%	−15.12%
	*p*-value*		**< 0.001**	**< 0.001**	**< 0.001**

*p-values of less than 0.05 (**in bold**) were counted statistically significant

*NYS* Nystatin, *FLU* Fluconazole, *MCF* Micafungin

Biofilm formation was detected in almost 22% (*n* = 7) of the tested isolates, showing variable levels of biofilm forming capacities. Isolates were further classified according to their capacities to produce biofilms. Almost 78% (*n* = 25) of the isolates were non-biofilm producers. On the other hand, two (6.2%), four (12.5%) and one (3.1%) isolates showed weak, moderate and strong biofilm formation abilities, respectively.

In order to determine if antifungal agents could affect biofilm production, biofilm producing isolates were subjected to sub-MICs of the tested antifungal agents and results are summarized in Table 2. There was a significant difference (*p* < 0.05) in OD_590_ values demonstrated by the control (untreated) and test *C. albicans* isolates (treated). The median OD_590_ value of the biofilm formation capacities of the control *C. albicans* isolates was 0.256 (range 0.1555–1.1165). Exposure to nystatin and micafungin significantly (*p* <0.05) reduced biofilm production with median OD_590_ values of 0.177 (30.86% reduction) and 0.182 (28.91% reduction), respectively. Inhibition of biofilm production after fluconazole exposure was insignificant (*p* > 0.05) in comparison to the untreated control (16.41% reduction) with median OD_590_ of 0.214. An additional table shows these results in more details [Additional file 2].

**Table 2:** Effect of antifungal subinhibitory concentrations on biofilm production (OD_590_) of *C. albicans* biofilm- producing isolates

Biofilm formation	Control	NYS	FLU	MCF
Median	0.256	0.177	0.214	0.182
Range	0.1555–1.1165	0.116–0.869	0.135–1.003	0.125–0.906
% Reduction		−30.86%	−16.41%	−28.91%
*p*-value*		**< 0.001**	0.884	**0.023**

*p-values of less than 0.05 (**in bold**) were counted statistically significant

*NYS* Nystatin, *FLU* Fluconazole, *MCF* Micafungin

**Fig. 1:**
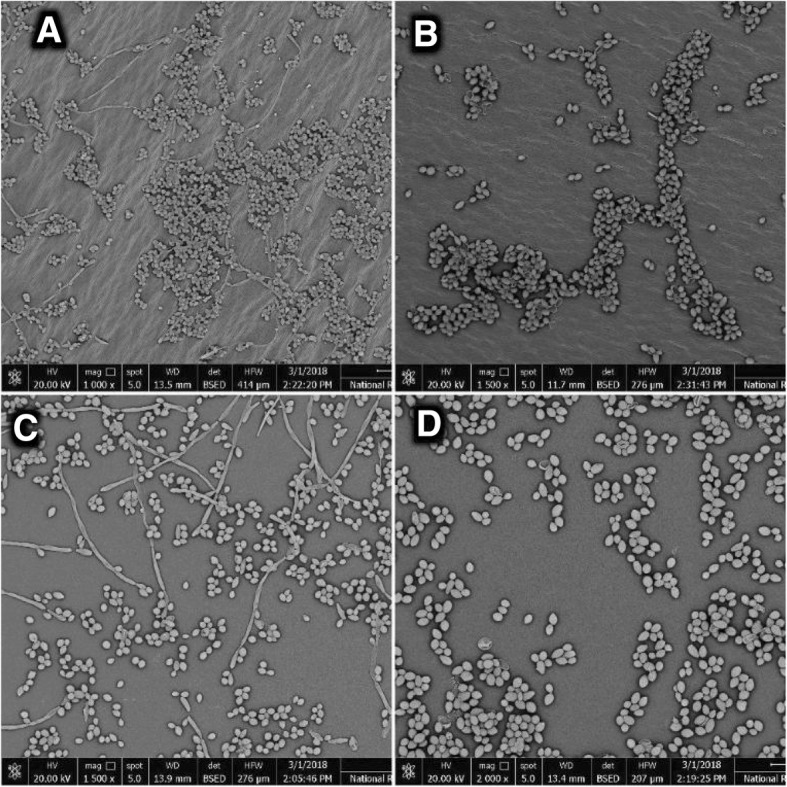
**a** Scanning electron micrograph for the untreated *C. albicans* isolate (control) showing yeast and hyphae in biofilm network. **b** Scanning electron micrograph for the nystatin-treated *C. albicans* isolate showing less dense biofilm network and arrested filamentation. **c** Scanning electron micrograph for the fluconazole-treated *C. albicans* isolate showing collapsed filaments. **d** Scanning electron micrograph for the micafungin-treated *C. albicans* isolate exhibiting loosening of cells and disappearance of hyphae

As shown in Fig. 1, scanning electron micrograph (SEM) of the untreated control cells displays a network of blastoconidia and hyphae in a typical *C. albicans* biofilm three dimensional structure (Fig. 1a). An additional image shows these results in more details [Additional file 3]. The NYS-treated (Fig. 1b) and the MCF-treated (Fig. 1d) cells exhibited loosening of cells and disappearance of filamentation, as well as shrinkage and lysis of some exposed cells. An additional image file shows this in more detail [see Additional file 4]. The FLU-treated cells (Fig. 1c) showed a decreased number of Candidal cells, yet displaying some collapsed hyphae.

## Discussion

In recent years, interest concerning virulence attributes of *C. albicans* has considerably increased. At least in part, this is due to discovering some drugs that may modulate their manifestation [36, 37]. Fungal infections caused by *C. albicans* have become more frequent and significant within few decades, causing a wide spectrum of superficial and deep infections [38], resulting in a serious public health issue medically and economically due to the high mortality rates, increased costs of therapy and duration of hospitalization [39, 40].

Candidiasis can be treated with polyenes, azoles and echinocandins. However, once administered, the concentration of these drugs tend to be reduced due to drug metabolism, tissue bioavailability, or the presence of biofilms [41]. Therefore, the impact of some commonly prescribed antifungal agents representing different classes and mechanisms of action, namely NYS (polyenes), FLU (azoles) and MCF (echinocandins) at their sub-MICs (0.5 x MIC) on various hydrolases and biofilm production by *C. albicans* isolates was investigated in the current study.

The current study’s observations revealed a significant (*p* < 0.05) inhibition of extracellular hydrolytic activities after being subjected to sub-MICs of antifungal treatment (NYS, FLU and MCF). Pertaining to phospholipase, the reduction in its production was proven to be the highest for NYS-(4.45% reduction), followed by MCF-(3.75% reduction) and then FLU-exposed yeasts (3.46% reduction). The present study’s results concerning the inhibition of phospholipase activity are, to a great extent, in accordance with the work of other investigators [22], who claimed that exposure to amphotericin B and nystatin had a significant inhibitory effect on the phospholipase production in clinical *C. albicans* isolates with a % reduction of 12.72 and 7.46, respectively. However, exposure to fluconazole reduced phospholipase activity insignificantly in the same study. In another study conducted by Ellepola et al. [12], they reported that exposure to nystatin, amphotericin B, caspofungin and ketoconazole resulted in a significant inhibition in phospholipase production with mean reduction percentages of 10.65, 12.14, 11.45 and 6.40, respectively, while exposure to fluconazole had an insignificant impact on phospholipase activities as compared to the unexposed control. These differences could be attributed to variations among test strains and source of clinical specimens as seen in the aforementioned studies of Anil et al. [22], whose isolates were collected from the oral cavity of HIV-infected individuals in Hong Kong and Ellepola et al. [12] whose oral isolates were obtained from different patients groups and healthy individuals in Kuwait.

Considering aspartyl protease, the reduction in proteolytic activity was the highest for NYS-(16.41% reduction), followed by MCF-(16.22% reduction) and then FLU-exposed yeasts (15.63% reduction). The present study’s findings regarding aspartyl protease inhibition through exposure to antifungals’ sub-MICs reflected some similarities with a previously reported study by Wu et al. [23] in Hong Kong, where they claimed that exposure to polyenes and imidazoles sub-MICs resulted in aspartyl protease reduction, indicating that antifungal therapy may modulate the proteolytic activity of pathogenic *C. albicans* recovered from HIV-infected and HIV-uninfected patients. They highlighted that aspartyl protease production was less inhibited by exposure to antifungal agents in the group of HIV-infected patients in comparison to the uninfected one, suggesting that *C. albicans* has more pathogenic potential in case of immuno-compromised patients.

Concerning haemolysin, the inhibition of haemolytic activity was recorded the highest for MCF-(15.12% reduction), followed by FLU-(13.65% reduction) and then NYS-exposed yeasts (12.36% reduction). The current study’s results concerning suppression of haemolysin production show some similarities with the work of previous investigators [11], where they reported a significant reduction in *C. albicans* and *C. tropicalis* haemolytic activity of isolated from HIV-positive patients upon exposure to sub-MICs of nystatin, amphotericin B and fluconazole [11], where the greatest haemolysin suppressive effect was detected in yeast cells exposed to amphotericin B, followed by nystatin and fluconazole. This disparity could be due to the fact that different clinical species and strains were used, in addition to the fact that echinocandins were not involved in the study of Anil et al. [11]. In another study reported by Negril et al. [42], they proposed that different *Candida* spp. demonstrated either reduced or increased haemolytic activities after being exposed to amphotericin B resolving to the conclusion that haemolysin activities were strain and species dependent without any association between activity profile and the site of isolation.

It could be debated that the investigated enzymatic activities in the current study are pertained to the Candidal offspring and not the parent cells subjected to the antifungal treatment. Nevertheless, the fact persisted is that exposure to sub-cidal concentrations of antifungals persevered into the succeeding generation thus affecting a key virulence attribute of the pathogenic fungus. Accordingly, all *C. albicans* isolates exposed to subinhibitory antifungal concentrations showed a significant (*p < 0.05*) reduction in phospholipase, aspartyl protease and haemolysin activities. The previous studies could not provide a precise explanation for the lowered hydrolytic enzyme activities by the antifungal-exposed yeasts, although the varying effects of polyenes, azoles and echinocandins on the yeasts’ plasma membrane ergosterol, the ergosterol synthesis and the cell wall β-D glucan synthesis respectively, may partly explain these observations.

Concerning inhibition of biofilm production in the presence of subinhibitory concentrations of the tested antifungal agents, the present study reflected more promising significant (*p < 0.05*) effects for treatment with NYS (30.86% reduction) and MCF (28.91% reduction), which are in accordance with studies reported by other investigators [43], who concluded that echinocandins and polyenes, represented by caspofungin and amphotericin B respectively, had the most optimum anti-biofilm activities, when compared to the observations of another study reported by Khan et al. [44], where fluconazole, the control drug of the study, showed a less effective capacity in preventing biofilm production. This observation comes in accordance with the current study’s results concerning the insignificant FLU suppressive effect on biofilm production by the test biofilm-producing isolates. These observations concerning FLU anti-biofilm activity may be attributed to the different mechanisms of activities of the antifungals incorporated in the study. Since fluconazole is fungistatic in action, this fact may render it with compromised biofilm-inhibiting abilities, in comparison to the other antifungal agents (NYS and MCF), which exert fungicidal effect, resulting in a more augmented anti-biofilm effects. It may also be pertained to the resistance pattern of the tested isolates. Since all the present study’s isolates showed high resistance rates to FLU, this could play a role in the weak FLU anti-biofilm activity, owing to the upregulation of the major facilitator superfamily (MFS) proteins and ATP binding cassette (ABC), contributing to the development of Candidal tolerance to antifungals [45].

In the present study, SEM observations showed intact biofilm network of yeast and hyphal cells by the untreated control clinical isolate, whereas treated cells revealed disorganization of biofilm structure. NYS-treated biofilm showed internally deflated cells with undamaged cell wall “ghosts-like” cells following exposure to sub-cidal concentrations of polyenes, which are in accordance with a study reported by other investigators [46]. Furthermore, subjecting *Candida* cells to MFC sub-MIC led to less dense biofilm network, which can be explained by the observations of a study reported by [47], where they claimed that low concentrations of MCF had an excellent inhibitory effect on the growth of *Candida* cells. FLU-treated cells were seen in scattered colonies with few filamentation. In a previous study conducted by Khan et al. [44], they reported that fluconazole showed less efficiency in preventing formation of Candidal biofilms. This could be attributed to that fact that it is fungistatic in nature, which leads to less anti-biofilm activity in comparison to the aforementioned counterparts (NYS and MCF).

## Conclusions

This study addressed the influence of subinhibitory concentrations of different antifungal agents on the pathogenicity of *Candida albicans*, represented in extracellular hydrolases and biofilm production, implying that exposure to commonly prescribed polyenes, azoles and echinocandins can modulate *C. albicans* pathogenicity. Hence, it can be claimed that antifungal agents have a dual beneficial effect by compromising virulence of the opportunistic fungus as well as killing or arresting its growth, which could potentiate the immune response towards eliminating *C. albicans* infections. Further genetic and molecular studies are necessitated to address the clinical relevance of the suppression of *Candida* hydrolytic enzymes activities and biofilm production in the presence of sub-MICs of antifungal treatment. This study would also encourage the development of new combination therapeutic plans utilizing antifungal agents of different classes and mechanisms of action, which can have a promising impact on lowering the escalating rates of antifungal resistance.

## Additional files

Additional file 1: Minimum inhibitory concentration values amongst *C. albicans* isolates for each antifungal agent. Description of data: Minimum inhibitory concentration (MIC) values (μg/mL) obtained by in vitro susceptibility testing using the broth microdilution method for *C. albicans* isolates. (XLSX 8 kb)

Additional file 2: Optical density (OD_590_) values of the control, nystatin, fluconazole and micafungin- exposed *C. albicans* biofilm- producing isolates. Optical density (OD_590_) values of the control (No drug), nystatin, fluconazole and micafungin- exposed (0.5 × MIC) *C. albicans* biofilm- producing isolates. (XLSX 10 kb)

Additional file 3: Scanning electron micrograph for the No- yeast control. A scanning electron micrograph showing the No- yeast negative control. (DOCX 396 kb)

Additional file 4: Scanning electron micrograph for the nystatin- treated *C. albicans* isolate. Scanning electron micrograph for the nystatin- exposed *C. albicans* isolate showing shrinkage and lysis of some exposed cells (ghost-like cells). (DOCX 652 kb)

## Abbreviations

CFU: Colony forming unit
CLSI: Clinical and Laboratory Standards Institute
FLU: Fluconazole
MCF: Micafungin
MICs: Minimum inhibitory concentrations
MOPS: N- Morpholinepropanesulfonic acid
NYS: Nystatin
OD: Optical density
PBS: Phosphate buffered saline
SDA: Sabouraud’s dextrose agar
SEM: Scanning electron microscopy/micrograph
sub-MICs: Subinhibitory concentrations
