# Supplementary figures and images for: Influence of subinhibitory antifungal concentrations on extracellular hydrolases and biofilm production by Candida albicans recovered from Egyptian patients
Source: BMC Infect Dis. 2019 Jan 16;19:54. doi: 10.1186/s12879-019-3685-0 (PMC6335770; doi:10.1186/s12879-019-3685-0)

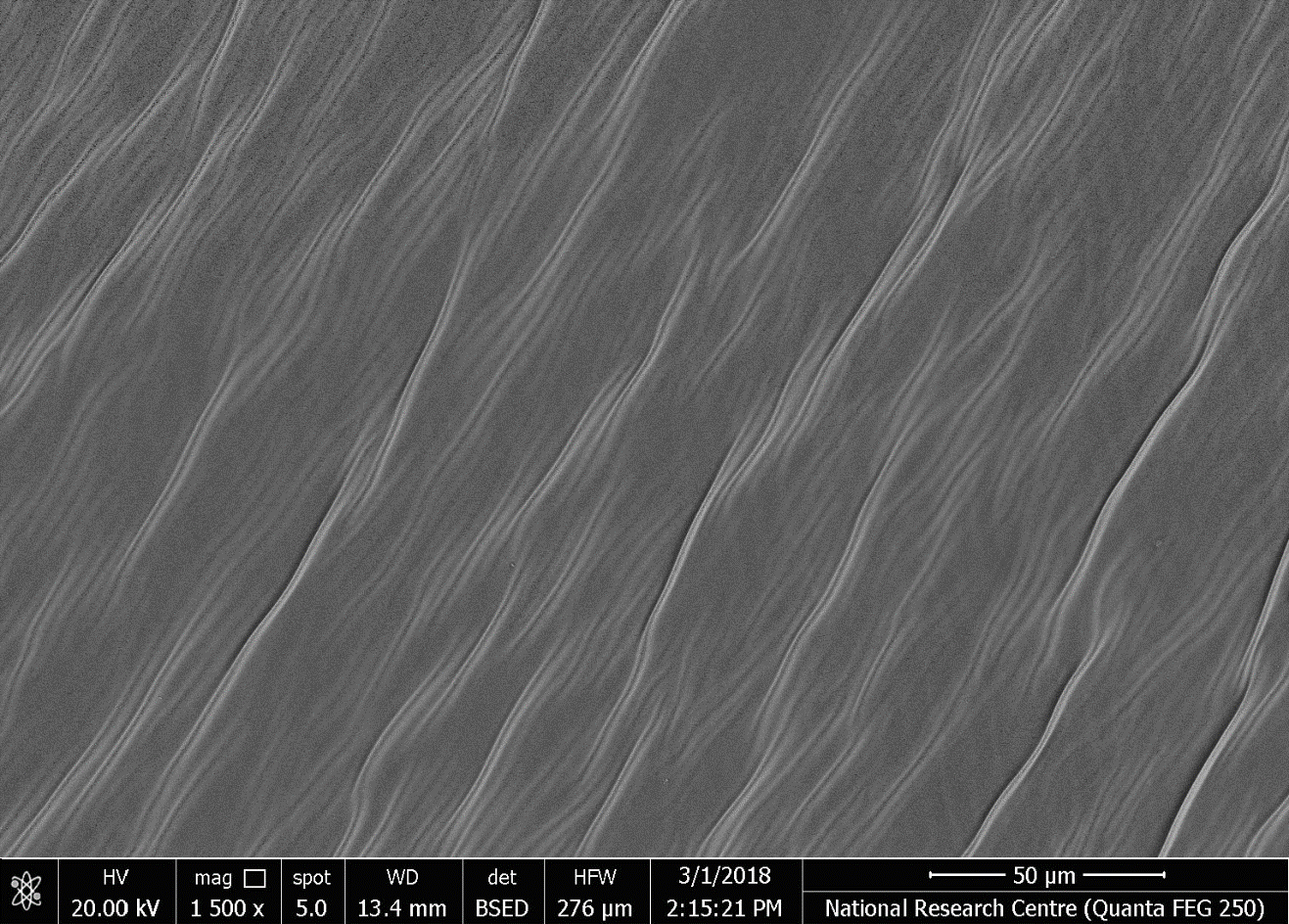

Supplement: Supplementary file 3 — Scanning electron micrograph for the No- yeast control. A scanning electron micrograph showing the No- yeast negative control. (DOCX 396 kb) [file 12879_2019_3685_MOESM3_ESM.docx]

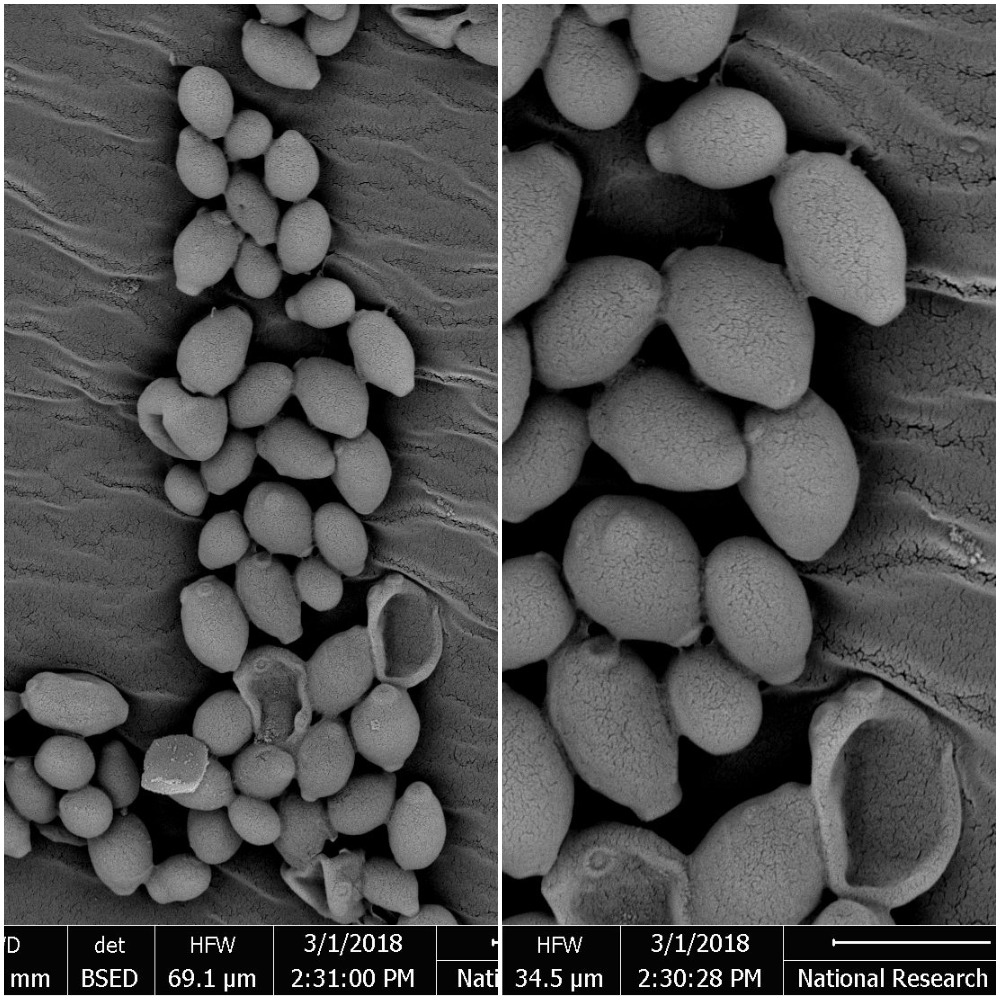

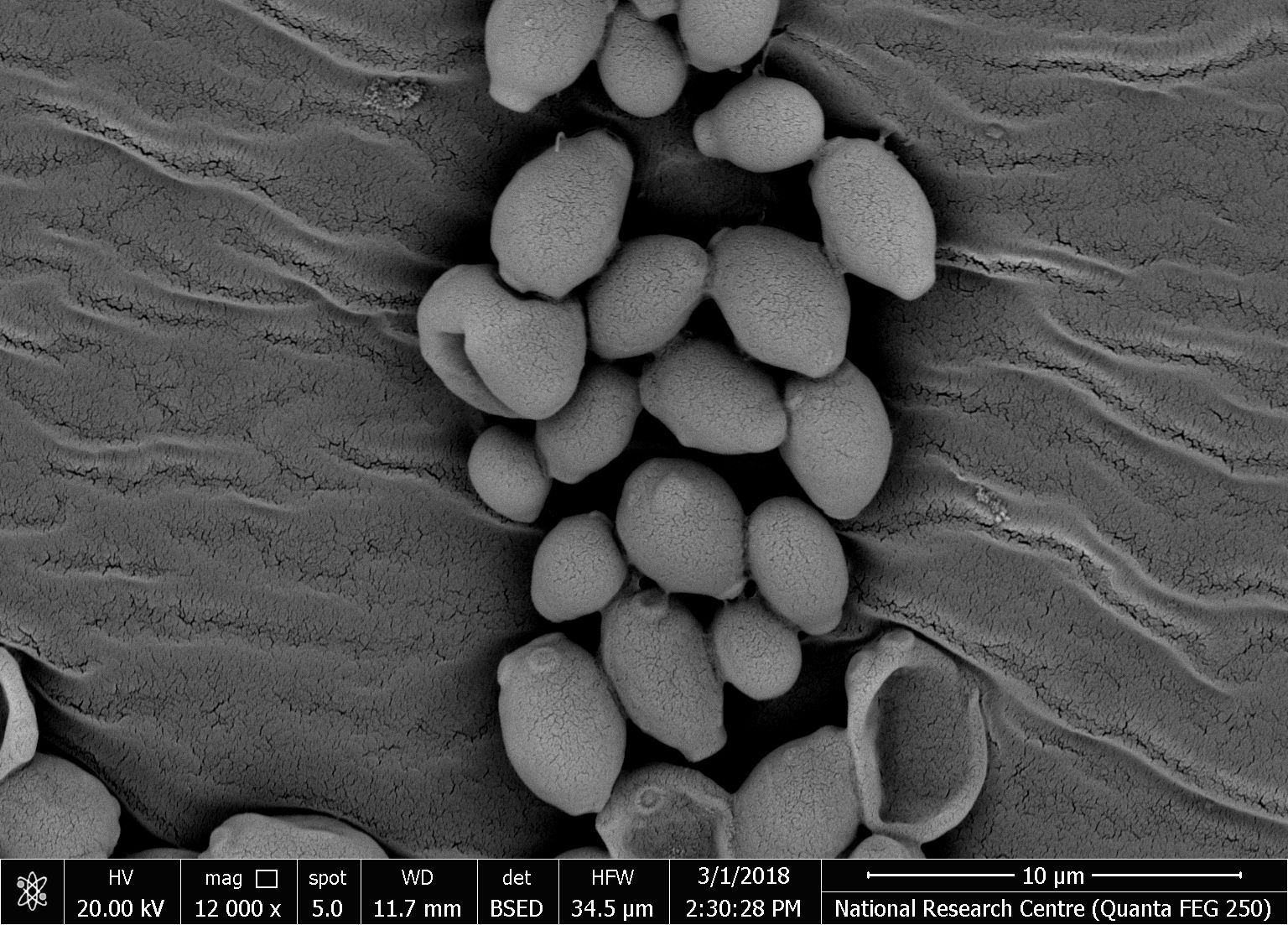

Supplement: Supplementary file 4 — Scanning electron micrograph for the nystatin- treated C. albicans isolate. Scanning electron micrograph for the nystatin- exposed C. albicans isolate showing shrinkage and lysis of some exposed cells (ghost-like cells). (DOCX 652 kb) [file 12879_2019_3685_MOESM4_ESM.docx]
